# Coupling brain-machine interfaces with cortical stimulation for brain-state dependent stimulation: enhancing motor cortex excitability for neurorehabilitation

**DOI:** 10.3389/fnhum.2014.00122

**Published:** 2014-03-05

**Authors:** Alireza Gharabaghi, Dominic Kraus, Maria T. Leão, Martin Spüler, Armin Walter, Martin Bogdan, Wolfgang Rosenstiel, Georgios Naros, Ulf Ziemann

**Affiliations:** ^1^Division of Functional and Restorative Neurosurgery, Division of Translational Neurosurgery, Department of Neurosurgery, Eberhard Karls University TuebingenTuebingen, Germany; ^2^Neuroprosthetics Research Group, Department of Integrative Neuroscience, Werner Reichardt Centre, Eberhard Karls UniversityTübingen, Germany; ^3^Department of Computer Engineering, Wilhelm-Schickard Institute for Computer Science, Eberhard Karls University TuebingenTuebingen, Germany; ^4^Department of Computer Engineering, University of LeipzigLeipzig, Germany; ^5^Department of Neurology and Stroke, Hertie Institute for Clinical Brain Research, Eberhard Karls University TuebingenTuebingen, Germany

**Keywords:** brain state-dependent stimulation, activity-dependent stimulation, closed-loop stimulation, brain-computer interface, brain-machine interface, brain-robot interface, transcranial magnetic stimulation, neurorehabilitation

## Abstract

Motor recovery after stroke is an unsolved challenge despite intensive rehabilitation training programs. Brain stimulation techniques have been explored in addition to traditional rehabilitation training to increase the excitability of the stimulated motor cortex. This modulation of cortical excitability augments the response to afferent input during motor exercises, thereby enhancing skilled motor learning by long-term potentiation-like plasticity. Recent approaches examined brain stimulation applied concurrently with voluntary movements to induce more specific use-dependent neural plasticity during motor training for neurorehabilitation. Unfortunately, such approaches are not applicable for the many severely affected stroke patients lacking residual hand function. These patients require novel activity-dependent stimulation paradigms based on intrinsic brain activity. Here, we report on such brain state-dependent stimulation (BSDS) combined with haptic feedback provided by a robotic hand orthosis. Transcranial magnetic stimulation (TMS) of the motor cortex and haptic feedback to the hand were controlled by sensorimotor desynchronization during motor-imagery and applied within a brain-machine interface (BMI) environment in one healthy subject and one patient with severe hand paresis in the chronic phase after stroke. BSDS significantly increased the excitability of the stimulated motor cortex in both healthy and post-stroke conditions, an effect not observed in non-BSDS protocols. This feasibility study suggests that closing the loop between intrinsic brain state, cortical stimulation and haptic feedback provides a novel neurorehabilitation strategy for stroke patients lacking residual hand function, a proposal that warrants further investigation in a larger cohort of stroke patients.

## Introduction

Despite intensive rehabilitation training according to evidence-based guidelines, functional restoration in patients with severe and persistent motor deficits following stroke is very limited (Kwakkel et al., 2003). Investigational studies are therefore currently examining different brain stimulation protocols at both ipsi- and contralesional stimulation sites, either as the only treatment modality or concurrent with traditional neurorehabilitation. Although results varied, when a combination of brain stimulation and motor training was applied, the probability of long-term neuroplastic changes according to Hebbian mechanisms improved (Edwardson et al., 2013).

Increasing excitability of ipsilesional primary motor cortex (M1) by transcranial magnetic stimulation (TMS) is one of the most explored approaches to prime use-dependent plasticity before motor exercises. Continuous stimulation with frequencies between 3 and 20 Hz (Khedr et al., 2005; Malcolm et al., 2007; Chang et al., 2010) and intermittent theta-burst protocols with 2 s-trains of short 50 Hz bursts (Talelli et al., 2012; Hsu et al., 2013) have both been applied to increase ipsilesional M1 excitability *prior to* neurorehabilitative training. In a complementary experiment, Koganemaru et al. (2010) applied 8 s trains of repetitive 5 Hz TMS *alternating* with 50 s periods of neurorehabilitative training. All these approaches used pre-defined stimulation parameters independent of the actual behavioral state of the patient.

Unlike these open-loop approaches, activity-dependent paradigms using neural or muscle activity to control brain stimulation in a closed-loop approach hold promise to invoke Hebbian mechanisms for more efficacious plasticity induction (Edwardson et al., 2013). Bütefisch et al. (2011) introduced activity-dependent TMS to stroke rehabilitation, in which motor activity-related electromyography (EMG) of the paretic limb triggered a single TMS pulse to the hand representation of the ipsilesional M1 *during* the motor exercises. Notably, the patients in that study—as in most other stroke studies on brain stimulation—suffered from mild to moderate paresis and were able to perform hand movements targeted by stimulation. Patients with severe hand paresis would be unlikely to benefit from such activity-dependent stimulation approach due to variable, decreased, or even missing voluntary EMG activation. These patients require novel activity-dependent stimulation paradigms based, for example, on intrinsic brain activity. Such alternative concepts have already been implemented in animal studies by using action potentials of single neurons to trigger electrical stimulation (Jackson et al., 2006; Rebesco et al., 2010), but have not been introduced for patients with motor deficits.

Volitional control of cortical neural activity in the absence of actual movements has been explored extensively to operate cursors or peripheral devices in the context of brain-computer (BCI)/brain-machine-interfaces (BMI) (Wolpaw et al., 2002). More specifically, electroencephalography (EEG)-based neurofeedback training with a BMI has been used to provide haptic feedback for motor rehabilitation of severely affected stroke patients (Ramos-Murguialday et al., 2013). Due to methodological limitations, such as stimulus noise contamination on cortical signal processing, these techniques have not yet included concurrent brain-stimulation applications. Walter et al. (2012) recently introduced the use of the Burg algorithm to minimize the influence of stimulation after-effects on spectral estimation of cortical signals for BMI.

In the present feasibility study, we investigated the effects of coupling a BMI with TMS, i.e., applying brain-state dependent stimulation (BSDS), on cortical physiology in the healthy and post-stroke brain. More specifically, we compared four experimental conditions in the healthy subject: (1) BSDS + HF, i.e., TMS was applied to the M1 hand area during event-related desynchronization in the ß-range (ß-ERD) induced by motor imagery, and combined with haptic feedback (HF) delivered by passive hand opening through a robotic hand orthosis; (2) HF alone; (3) non-specific brain stimulation (NSBS), i.e., TMS without motor imagery or HF; and (4) NSBS + HF.

We hypothesized—and confirm here—that BSDS + HF induced the most pronounced use-dependent cortical increase in corticospinal excitability and M1 hand representation. In a second step, we explored the feasibility and accumulative effect of this novel BSDS + HF paradigm in a severely affected stroke patient with no residual finger extension. In particular, we wished to ascertain the extent to which the absence of motor evoked responses (MEP > 50 μV) at baseline could be turned into the presence of MEP in the ipsilesional M1 hand representation.

## Materials and methods

### Subjects

Two participants (one healthy 24-year-old female subject; one 41-year-old, male patient, who suffered a right subcortical/cortical ischemic stroke 5 years ago and who has a persistent severe left-sided hemiparesis (upper extremity Fugl-Meyer score of 9) and no voluntary control of finger extension, Medical Research Council scale < 2) without contraindications to TMS, gave written informed consent before participating in this study, which was approved by the local ethics committee. We traced changes in MEP amplitude of the extensor digitorum communis (EDC) muscle following different intervention protocols. The healthy participant was subjected to one single session each of four different experimental conditions (brain state-dependent stimulation (BSDS) and three control conditions reported in detail below), which were carried out at intervals of at least 11 days. These single experimental conditions were performed to disentangle the respective contributions of brain stimulation and haptic feedback to plastic changes of the motor cortex. The patient underwent 20 identical sessions of only one of the conditions (BSDS) on consecutive working days over a period of 4 weeks. Thereby, we intended to demonstrate the feasibility of BSDS *during* motor exercises of a neurorehabilitation program, specifically for severely affected stroke patients who are unable to actively engage their affected hand in rehabilitation exercises. The study outcomes were recorded before and after the single sessions of each condition in the case of the healthy control; and before and after the 20 sessions in the case of the stroke subject.

### TMS mapping protocol

For mapping and stimulation during the intervention, we used a navigated TMS stimulator (eXimia^®^ NBS, Nexstim, Helsinki, Finland), with a figure-8 biphasic eXimia Focal Bipulse Coil (5 cm winding diameter) and coregistered individual anatomical T1 weighted magnetic resonance images (acquired with 3-Tesla TIM-TRIO-system, Siemens AG, Germany). Participants were seated in a comfortable reclining chair for the duration of the mapping and the following intervention. The representation of the left EDC, eliciting the largest MEP (“hotspot”) in the right M1, was determined using a standard mapping protocol and a coil orientation perpendicular to the central sulcus (Wassermann et al., 2008). The “hotspot” thus resolved upon was set as a stimulation point and remained constant during all experiments. Before and after each intervention, we acquired a MEP stimulus-response curve (SRC) and a cortical map representation at 110% resting motor threshold (RMT, defined as minimum stimulus intensity to result in at least 5/10 MEPs > 50 μV) in the healthy subject. The SRC was acquired with 10 stimuli each at 40, 50, 60 and 70 V/m of the calculated electrical field, respectively, and these intensities were expressed as percentage of the resting motor threshold. The cortical map in the patient was acquired at maximum stimulator output with three stimuli per cell. For both healthy subject and patient, the cell size of the virtual grid was 5 × 5 mm and the total grid size was 10 × 10 cm covering the primary motor cortex, the somatosensory cortex, the premotor cortex and the supplementary motor area. During all TMS measurements, participants were requested to keep their muscles relaxed. All trials were visually inspected offline and rejected from analysis where muscle pre-activation was present (<1% of cases). Electromyographic (EMG) activity was recorded using Ag/AgCl surface electrodes (Ambu Neuroline720, Ambu GmbH, Germany), placed 2 cm apart on the muscle belly. During the intervention period, a BrainAmpExG-Amplifier (Brainproducts GmbH, Germany) with 1 kHz sampling rate, high-pass filtering at 0.16 Hz and low-pass filtering at 1000 Hz, was used to record EMG activity. For the TMS mapping before and after the intervention, the integrated 6-channel EMG device of the TMS system was used with 3 kHz sampling rate and a band-pass filter of 10–500 Hz.

### Experimental set-up

**Figure 1:**
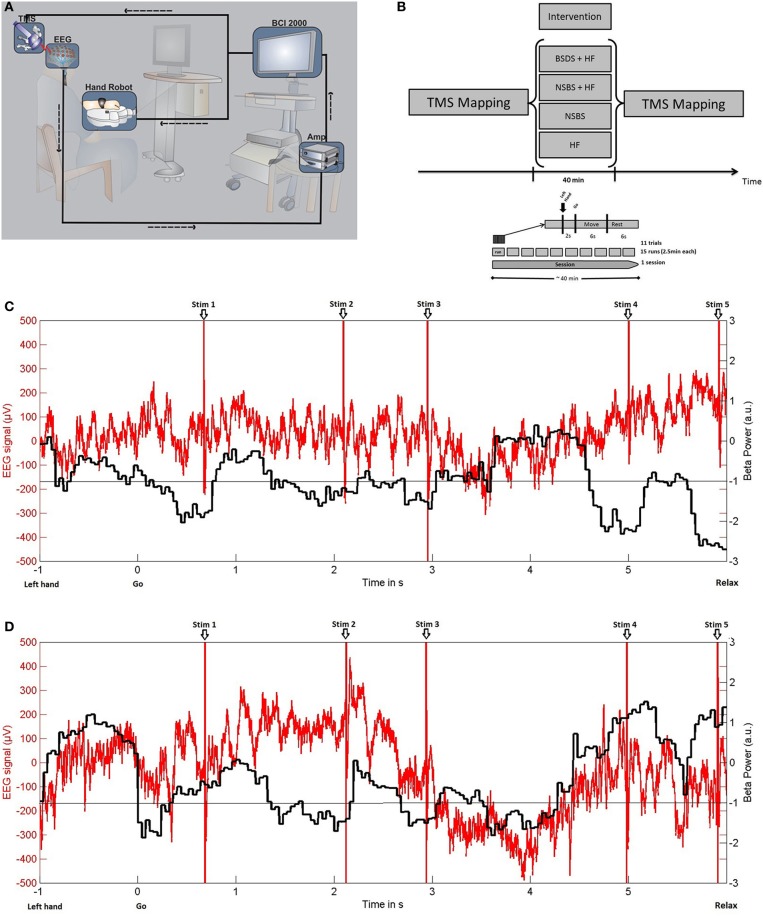
**(A)** Experimental set-up for brain-state dependent stimulation (BSDS) including electroencephalography (EEG) recording, signal amplification (Amp), software for online analysis, and triggering of haptic feedback (hand robot) and transcranial magnetic stimulation (TMS) within a closed-loop framework. **(B)** Study design. **(C)** BSDS + HF condition: (−1). Exemplary single trial raw data of EEG recordings (red) from electrode C4 of the healthy subject (in μV, left y-axis) and the online classifier output (black, ß-power, in arbitrary units, right y-axis). Please note that, in this closed-loop feedback condition, motor imagery-related ERD frequently reaches the pre-defined threshold during the movement imagination phase (6 s after “go” signal). Moreover, TMS is applied during these ERD phases only. **(D)** NSBS + HF condition: Exemplary single trial demonstrating that TMS with timing replayed from the BSDS + HF condition trial shown in **(C)** is applied independently of ERD. Otherwise, the same conventions as in **(C)** apply.

We implemented a closed-loop set-up, where TMS of the EDC representation of the right M1 (hand opening) was triggered online by oscillatory brain activity during cued kinesthetic motor imagery of hand opening (Figure 1A).

Oscillatory brain activity was recorded in a 32-channel EEG set-up according to the extended 10–20 system using Ag/AgCl electrodes and BrainVision software with DC-Amplifiers (BrainAmp, Brainproducts GmbH, Germany). All impedances were maintained below 10 kΩ. Following digitization at 1 kHz rate, high-pass filtering with 0.16 Hz and low-pass filtering with 1000 Hz, the EEG signals were transferred to BCI2000 software (Schalk et al., 2004) for online analysis, triggering, and offline storage.

Participants were asked to imagine how it feels to open their left hand without actually doing so. Each experiment consisted of 15 runs, each lasting approximately 2.5 min. Each run consisted of 11 trials. Trials commenced with a preparation phase of 2 s, followed by a 6 s movement imagination phase and a 6 s rest phase (Figure 1B). The subjects were asked to imagine opening their hand in each trial synchronous to the movement of the robotic hand orthosis. Preparation and imagination phases were initiated by the auditory cues “left hand” and “go” respectively, which were audiotaped words of a female voice.

A robotic hand orthosis (Amadeo^®^, Tyromotion GmbH, Austria) passively opened the attached left hand whenever motor imagery related event-related desynchronization (ERD) in the beta-band (16–22 Hz) was detected during the movement imagination phase (Figure 1C) on three electrodes over the right sensorimotor area (FC4, C4, CP4). This set-up provided haptic feedback to successful volitional modulation of oscillatory brain activity, thereby facilitating the decoding of motor imagery related ERD.

Simultaneously, BSDS was applied with a single TMS pulse to the EDC “hot-spot” of the right motor cortex, at 110% RMT for the healthy subject and at 100% stimulator output for the stroke patient, triggered by BCI2000 whenever detecting motor-imagery related beta-ERD. The TMS stimuli were triggered when a minimum of 200 ms of consecutive ERD was detected. The interval between two TMS pulses was at least 500 ms. This resulted in an average of 1.78 ± 0.48(*SD*) pulses per trial and a total of 293 pulses during one experimental condition. Stimulation ceased once ERD disappeared. ERD detection was performed with an adaptive linear classifier. In order to detect the ERD, we extracted the spectral power values between 16 and 22 Hz in three bins of width 2 Hz for each of the channels FC4, C4 and CP4, resulting in nine input features. The spectral power was computed with an autoregressive model order of 16 (McFarland and Wolpaw, 2008), fitted to the last 500 ms of the signal and updated every 40 ms. To avoid a noisy control signal for the orthosis and the TMS device, five consecutive 40 ms epochs (i.e., 200 ms) had to be classified as ERD positive (negative) in order to start (stop) stimulation. An epoch was classified as ERD-positive only if the output of the classifier exceeded a threshold. This threshold was individually determined from three training runs to ensure that feedback and stimulation in the test sessions were provided only when the subjects reached 20% of their strongest ERD modulation. A period of 50 ms was removed after every pulse to prevent contamination of the brain signal due to the stimulation artifact. The resulting signal with gaps was analyzed using a modified Burg algorithm for segmented data in the online analysis (Walter et al., 2012).

In the healthy subject we examined four experimental conditions (Figure 1B): (a) HF alone (BMI with haptic feedback alone, with motor imagery) (b) NSBS (repetitive-like TMS alone, no motor imagery) (c) NSBS + HF (combination of BMI with haptic feedback and repetitive-like TMS, with no motor imagery) (d) BSDS + HF (with motor imagery). In NSBS + HF and NSBS, the healthy subject was requested to maintain muscle relaxation during the intervention, i.e., not to perform the motor imagery task, as motor imagery would induce ERD modulation as in the BSDS condition. The sequence of TMS pulses was recorded in the first experiment (BSDS + HF) and then replayed in exactly the same way in the NSBS experiments (Figure 1D). This ensured that precisely the same stimulation pattern and intensity were applied at the identical site in the NSDS experiments, but in a random manner with regard to the ongoing brain activity. Thus, for all the conditions, the same stimulation parameters were used as well as the same number of pulses and intensity of stimulation. The RMT was obtained for each single session of the four different experimental conditions. RMT did not vary across the different experimental conditions (36% ± 0.8 of maximum stimulator output), ensuring that the stimulation intensity was the same for all condition.

### Statistical analysis

For the healthy subject, rmANOVA was performed on the EDC MEP SRC with the main effects Time (pre/post), Intensity (four levels) and Experiment (four levels) followed by *post-hoc* two-sample *t*-tests. Mean map MEP was a calculated mean of MEP amplitudes across all TMS sites with MEPs > 50 μV in the healthy subject and with MEPs > 10 μV in the stroke patient. Changes to baseline were analyzed pre vs. post in all four conditions using a two-sample *t*-test. Map area with MEPs > 50 μV was analyzed descriptively. Due to a lack of MEPs > 50 μV in the stroke patient prior to BSDS, mapping data (mean map MEP and map area) were analyzed descriptively.

## Results

Identification of single-trial ERD during motor imagery was suitable for a brain activity-dependent transcranial stimulation protocol. The setup operated reliably during online-application in the healthy subject and the stroke patient with severe hand paresis and was tolerated well.

**Figure 2:**
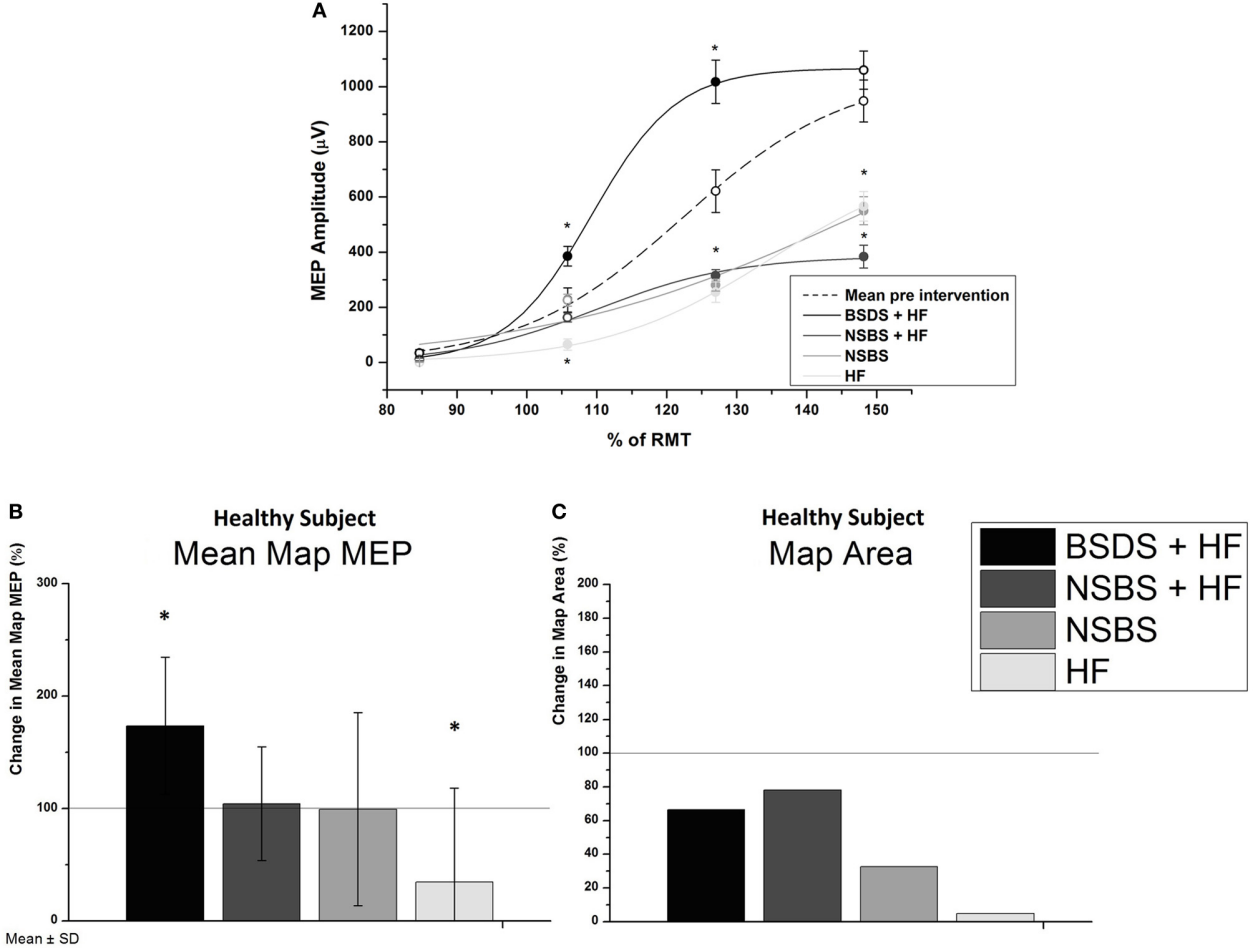
**(A)** MEP stimulus-response curves (SRC) for the healthy subject in all conditions compared to mean baseline curve of all experiments. Experimental interventions were brain-state dependent stimulation with haptic feedback (BSDS + HF), non-specific brain stimulation with (NSBS + HF) and without haptic feedback (NSBS), and haptic feedback without brain stimulation (HF). None of the baseline curves differed significantly from the mean baseline curve of all experiments. Significant differences, as determined by Bonferroni corrected two-sample *t*-tests (*p* < 0.05) of the post-intervention curve to baseline, are indicated by filled symbols and “∗.” Error bars indicate standard error of the mean (s.e.m.). **(B)** Changes in cortical map parameters due to intervention for the healthy subject (100% line indicating no change), mean map MEP ± SD and map area of all MEPs > 50 μV

In the healthy subject, BSDS significantly increased the EDC MEP SRC (*p* < 0.05), while all control conditions resulted in a decrease (all: *p* < 0.05), as shown by rmANOVA and *post-hoc* tests (Figure 2A). RmANOVA showed a significant difference in the factor Time (*F* = 17.29; *p* = 0.003) between baseline and post-intervention and between stimulus intensities (*F* = 882.5; *p* < 0.0001). Additionally, there was a significant difference between experiments (*F* = 100.26; *p* < 0.0001) and an interaction of Time and Intensity (*F* = 26.67; *p* = 0.001), of Time and Experiment (*F* = 47.28; *p* < 0.0001) and also between Intensity and Experiment (*F* = 18.52; *p* = 0.003). The interaction of Time × Intensity × Experiment was not significant (*F* = 3.552; *p* = 0.096). *Post-hoc* tests revealed a significant increase of MEP amplitude only in the BSDS + HF condition; all other conditions showed a significant decrease compared to baseline. Similar results were obtained for the EDC maps (Figure 2B), with a significant increase in the mean map MEP (*p* = 6.96e-4) following the BSDS + HF intervention and a significant decrease following the HF intervention (*p* = 0.006). Mean baselines in the healthy subject were 200.2 ± 76.5 μV for the map MEP, and 1194.2 ± 152.8 mm^2^ for the map area, respectively.

**Figure 3:**
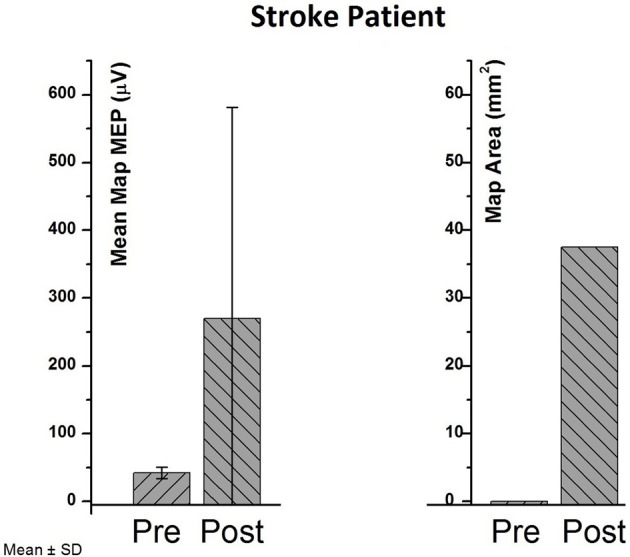
**Pre- vs. post-values in cortical map parameters (mean map MEP and map area) of the chronic stroke patient**.

In the stroke patient, no MEP SRC could be acquired, i.e., no resting motor threshold (5/10 MEPs > 50 μV) could be detected despite maximum stimulator output. Therefore, the TMS mapping (including MEPs < 50 μV) was used to capture a change. Repetitive application of the BSDS protocol resulted in MEPs > 50 μV in the post-measurement, and in an increase of both the average map MEP amplitude (42–269.6 μV) and of the MEP area (Figure 3). The upper extremity Fugl-Meyer Score changed from 9 to 10 following the intervention. This improvement was too small to result in a functionally meaningful motor improvement.

## Discussion

The specific timing of cortical stimulation has been identified as a critical factor that requires further investigation, with regard to both intrinsic brain activity and the respective rehabilitation exercise, for improving the consistency of brain stimulation effects during neurorehabilitation (Plow et al., 2009). In this context, movement-related TMS has been shown to induce motor plasticity in healthy subjects when applied at specific timing with respect to voluntary finger movements during a reaction time task (Thabit et al., 2010). A similar approach in stroke patients, namely the application of EMG-triggered TMS in strict temporal relationship to a wrist movement, showed obvious changes in motor maps but only subtle facilitatory effects on motor cortex excitability (Bütefisch et al., 2011). These findings are consistent with the Hebbian principle that long-term potentiation of synaptic efficacy that occurs when its pre- and post-synaptic elements are simultaneously active (Hebb, 1949).

Here, we have reported on a closed-loop TMS set-up that allowed for BSDS of motor cortex under direct volitional control of the stimulated subject. We demonstrated that such a BSDS paradigm is feasible in both healthy and brain-lesioned conditions. In addition, the robotic hand orthosis facilitated motor-imagery with contingent haptic feedback and enabled a patient with severe limb weakness to engage in rehabilitation exercises of finger extension during brain stimulation without residual finger movement. Closing the loop between intrinsic brain-state, cortical stimulation and haptic feedback thus increased the excitability of the stimulated M1 hand representation, and turned the absence of MEP (>50 μV) to a presence of MEP in the stroke patient. However, this increased excitability of the ipsilesional M1 was not paralleled by functional motor improvement, which might be explained by the limited length of the training period (Langhorne et al., 2009) and/or to the lesion location (subcortical/cortical). Ameli et al. (2009) have shown that only patients with a subcortical stroke improved in a motor task following ipsilesional high-frequency TMS. Moreover, brain stimulation protocols and neurorehabilitation outcome data on severely affected stroke patients with persistent deficits, such as the patient involved in the present study, are particularly rare and perspectives of motor restoration are *per se* limited (Koganemaru et al., 2010).

The size of muscle responses to TMS of the human M1 was recently shown to depend on pre-stimulation beta-band oscillatory activity of the cortex (Schulz et al., 2013) with ERD of the sensorimotor rhythm leading to increased MEP amplitudes (Takemi et al., 2013). Moreover, volitional control of this oscillatory activity during motor-imagery could be facilitated by haptic feedback (Gomez-Rodriguez et al., 2011). Our approach utilized and combined these findings by synchronizing brain stimulation with motor imagery-related desynchronization of oscillatory activity and haptic feedback, thereby reducing intrinsic variations of neuronal excitability at the time of stimulation and facilitating the induction of plastic changes.

The present BSDS paradigm applied single stimulation pulses with an average of two pulses per trial, i.e., during 6 s of motor imagery, thus presenting a completely different stimulation pattern than conventional theta-burst or high-frequency regular repetitive TMS paradigms that also aim to increase M1 excitability. Our approach enabled us to apply brain stimulation pulses *during* motor exercises and not *prior to* or *alternating with* neurorehabilitation training as is otherwise the case (Khedr et al., 2005; Malcolm et al., 2007; Chang et al., 2010; Koganemaru et al., 2010; Talelli et al., 2012; Hsu et al., 2013). Coupling the TMS pulses to ipsilesional ERD therefore ensured that intracortical connections targeting pyramidal tract neurons were stimulated when they were voluntarily depolarized through motor-imagery related ERD (Day et al., 1987; Bütefisch et al., 2011). As motor-imagery related ERD has been shown to reduce intracortical inhibition (Takemi et al., 2013) our approach modulated the susceptibility of these motor circuits to an excitatory drive. Accordingly, we applied motor imagery-related ERD as the pre-synaptic input, thereby fulfilling the requirements of Hebbian-stimulation (Hebb, 1949). Notably, motor-imagery related ERD with haptic feedback alone (without concurrent brain-state dependent cortical stimulation) led to a significant decrease of the motor cortex excitability, as did NSBS with and without haptic input (Figure 2). These findings suggest that the brain-state dependent coupling between central and peripheral input is essential for the observed plastic changes.

The combination with a BMI set-up moving the paralyzed hand contingent with motor intention also paves the way for the application of Hebbian-stimulation to many severely affected stroke patients who are unable to actively engage their affected hand in rehabilitation exercises (Kwakkel et al., 2003). The current results demonstrated the feasibility of BSDS to induce reorganization of the motor cortex. However, studies on a larger scale and with functional end points are necessary before the utility of this novel approach for stroke rehabilitation can be recognized.

### Conflict of interest statement

The authors declare that the research was conducted in the absence of any commercial or financial relationships that could be construed as a potential conflict of interest.
